# Effects of Arbuscular Mycorrhizal Fungi on Watermelon Growth, Elemental Uptake, Antioxidant, and Photosystem II Activities and Stress-Response Gene Expressions Under Salinity-Alkalinity Stresses

**DOI:** 10.3389/fpls.2019.00863

**Published:** 2019-07-03

**Authors:** Lin Ye, Xia Zhao, Encai Bao, Kai Cao, Zhirong Zou

**Affiliations:** ^1^College of Horticulture, Northwest A&F University, Xianyang, China; ^2^College of Agriculture, Ningxia University, Yinchuan, China; ^3^Key Laboratory of Agricultural Engineering in the Middle and Lower Reaches of Yangtze River, Ministry of Agriculture, Institute of Agricultural Facilities and Equipment, Jiangsu Academy of Agricultural Sciences, Nanjing, China

**Keywords:** *Citrullus lanatus* L., salinity-alkalinity conditions, antioxidant abilities, ROS scavenging enzymes, chlorophyll fluorescence, chloroplast ultrastructure

## Abstract

Salinity-alkalinity stress has caused severe environment problems that negatively impact the growth and development of watermelon (*Citrullus lanatus* L.). In this study, watermelon seedlings were inoculated with the arbuscular mycorrhizal fungi (AMF) *Funneliformis mosseae* to investigate its effect on watermelon growth and development. The main measurements included morphological traits, elemental and water uptake, the level of reactive oxygen species, antioxidant enzyme and photosynthesis activities, and relative expression levels of stress response genes. Under salinity-alkalinity stresses, watermelon morphological traits, elemental and water uptake were all significantly alleviated after incubation with AMF. Antioxidant abilities of watermelon were significantly improved after incubation with AMF in salinity-alkalinity stresses. Under normal conditions, all photosynthesis related parameters were significantly increased after incubation of AMF. In contrast, they were all significantly reduced under salinity-alkalinity stresses and were all significantly alleviated after incubation of AMF. Salinity-alkalinity stresses impacted the chloroplast structure and AMF significantly alleviated these damages. Under salinity-alkalinity stresses, the relative expression level of *RBCL* was significantly reduced and was significantly alleviated after AMF treatment. The relative expression level of *PPH* was significantly increased and was further significantly reduced after AMF treatment. For the relative expression levels of antioxidant response related genes *Cu-Zn SOD, CAT, APX, GR*, their relative expression levels were significantly increased and were further significantly increased after AMF treatment. Our study demonstrated the beneficial effects of AMF under salinity-alkalinity stresses, which could be implicated in the management of watermelon cultivation under salinity-alkalinity regions.

## Introduction

Soil salinization and alkalization have become major contributing factors to the ever increasing areas of land degradation worldwide, particularly in arid and semi-arid areas (Al-Karaki, 2006; Aragüés et al., 2015). Over 800 millions ha of soil resources worldwide are affected by soil salinity or alkalinity, representing 70% of the total agricultural land. High salt accumulations in plants lead to osmotic stress and ionic toxicity, thereby inhibiting plant growth by disrupting many physiological processes, leading to the decrease of crop productivity and quality (Evelin and Kapoor, 2014; Liu et al., 2016; Shamshiri and Fattahi, 2016).

The production of reactive oxygen species (ROS) is an important indicator for plants under salinity-alkalinity stress conditions (Noctor et al., 2014). High levels of ROS severely destroy cell membranes and impact a wide range of essential macro-molecules, such as photosynthetic pigments, proteins and DNA (Noctor et al., 2014; Li et al., 2017; Cao et al., 2018; Abdel Latef et al., 2019). Plant antioxidant systems can eliminate excess ROS produced under salinity stress by developing enzymatic and non-enzymatic compounds, such as superoxide dismutase (SOD), catalase (CAT), ascorbate peroxidase (APX), and glutathione reductase (GR) (Miller et al., 2010; Noctor et al., 2014; Pedranzani et al., 2016). Photosynthetic rate and carbon assimilation are significantly decreased under salinity-alkalinity stress (Hu et al., 2014). In contrast, the accumulation of ROS species is significantly increased (Li et al., 2017). Over accumulation of ROS inhibits the photosynthetic related enzyme activities and destroys the photosynthetic apparatus within the cell (Allakhverdiev et al., 2008).

To improve crop growth and stress tolerance under high salinity-alkalinity conditions, studies have focused on physical (water-conservation strategies), chemical (ameliorants for amendment), and biological (adopting salt-tolerant plants) remediation processes (Ravindran et al., 2007; Wang et al., 2014; Zhang et al., 2016). Recent researches showed that arbuscular mycorrhizal fungi (AMF) facilitate the salinity-tolerance of plants by forming mutualistic relationships with plants (Coleman-Derr and Tringe, 2014; Evelin and Kapoor, 2014; Lenoir et al., 2016; Liu et al., 2016; Shamshiri and Fattahi, 2016). AMF occurs extensively in saline soils with good bio-amelioration performances by improving nutrient (Estrada et al., 2013) and water uptake (Aroca et al., 2007), keeping high K^+^/Na^+^ ratio (Porcel et al., 2012), higher osmoprotectants (Garg and Baher, 2013), higher photosynthesis ability (Chen J. et al., 2017) and enzyme activities (Pedranzani et al., 2016).

Mitigative effects of AMF on plant growth and development under salinity-alkalinity conditions have been described in citrus (Wu et al., 2010), apple (Yang et al., 2014), olive (Porras-Soriano et al., 2009), wheat (Daei et al., 2009), soybean (Hashem et al., 2019), maize (Zhang et al., 2016), tomato and pepper (Abdel Latef and He, 2011; Abdel Latef and Chaoxing, 2014), cucumber (Hashem et al., 2018), and lettuce (Vicente-Sánchez et al., 2014). Watermelon (*Citrullus lanatus* L.), an economically important food worldwide, is usually considered as a high water-consuming and salinity-sensitive species. AMF colonization improves the watermelon yield and water use efficiency under water-deficiency conditions (Mo et al., 2016). In the present study, we aim to investigate the effects of AMF under salinity-alkalinity stresses on: (1) watermelon morphological development and elemental uptake; (2) the chlorophyll content, fluorescence, and ultrastructure as well the super oxidant species and antioxidant enzyme activities; (3) the relative expression levels of some important antioxidant response related genes, such as Cu-Zn subunit-superoxide dismutase (*Cu-Zn SOD*), catalase (*CAT*), cytoplasmic ascorbate peroxidase (*APX*), and cytoplasmic *GR*. This study will provide useful insights into the beneficial effects of AMF for watermelons and can also be applied to other species grown under salinity-alkalinity conditions.

## Materials and Methods

### Plant and Fungal Materials

The watermelon (*C. lanatus* L.) cultivar Fu Yunlai was obtained from the Shaanxi Yufeng Seed Industry Co., Ltd., Yangling, China. The AMF species *Funneliformis mosseae* (National Microbial Resources Platform number 1511C0001BGCAM0059) was obtained from the Institute of Plant Nutrition and Resources, Beijing Academy of Agriculture and Forestry Sciences, Beijing, China. The inoculum was prepared at the Northwest Agricultural and Forestry University, Yangling, China, by cultivation with *Zea mays* L. as the host plant in a 1:1 mixture of zeolite and sand. The prepared inoculum consisted of dried cultivation substrates containing spores (approximately 70 g^-1^), extraradical hyphae, and macerated *Z. mays* roots.

### Plant Growth Conditions and Experimental Design

Watermelon seeds were soaked in a 5% sodium hypochlorite for 10 min, and then thoroughly rinsed in running water, then directly sown on germination paper, and incubated at 25°C. After germination, seedlings were sown into commercial substrate in 13 × 13 × 8.5 cm polystyrene pots (Shaanxi Yufeng Seed Industry Co., Ltd., Yangling, China) (Li et al., 2015; Cao et al., 2018), and every treatment was consisted of 20 pots (each pot contained one watermelon plant) following a random combination. Prior to use, the substrate was sieved through a 4 mm mesh sieve and sterilized for 4 h at 160°C. The soil chemical properties were as follows: pH 7.16, organic matter 21.0%, and available N, P, and K 151.12, 61.27, and 258.46 mg⋅kg^-1^, respectively. At planting, half of the germinated seeds were inoculated with 10 g of the AMF inoculum per pot (mycorrhizal treatment); the other half received the same amount of inoculum sterilized by autoclaving (non-mycorrhizal treatment). Mycorrhizal inocula were placed directly below the watermelon seeds at the time of sowing (Mo et al., 2016). Plants were placed in an environmentally controlled greenhouse at Northwest Agriculture and Forestry University, Yangling, Shaanxi, China. The greenhouse was completely enclosed in glass and equipped with a pad-and-fan cooling system and water heating system. The environmental condition in the greenhouse was maintained at 25°C to 30°C during the day and 15°C to 18°C at night, with a relative humidity of 60 to 75% (Mo et al., 2016), during the period of March to May, 2015. The light intensity during the growing period ranged from 956 to 1378 μmol/(m^2^⋅s) and was measured using a spectroradiometer (Model PS-100, Apogee Instruments Inc., Logan, UT, United States). When watermelon plants had three true leaves, for both AMF-inoculated and non-inoculated plants, half of the plants were irrigated with 400 mL 60 mM salinity-alkalinity solution (NaCl: Na_2_SO_4_: NaHCO_3_: Na_2_CO_3_ = 1: 9: 9: 1; 80 mL per plant) (Hu et al., 2014; Li et al., 2015), and the other half received the same amount of water. The experimental plots included four treatments: (a) CK, without AMF inoculation + without salinity-alkalinity; (b) M, with AMF inoculation + without salinity-alkalinity; (c) S, without AMF inoculation + with salinity-alkalinity; and (d) MS, with AMF inoculation + with salinity-alkalinity. On day 6 after salinity-alkalinity treatment, leaf samples (the second fully expanded leaf beneath the growing point) were harvested after measuring the gas exchange and chlorophyll fluorescence parameters. Harvested samples were then rapidly frozen in liquid nitrogen and stored at -80°C until the biochemical assay.

### Determination of Mycorrhizal Colonization and Plant Morphological Traits

To determine the percentage mycorrhizal colonization of the root, 10 plants were randomly selected from each treatment. Each plant was considered as a biological replicate, and three measurements were carried out on each plant and the mean measurement was used as one biological replicate measurement. Mycorrhizal colonization was determined using the magnified root intersection method (McGonigle et al., 1990) after staining with trypan blue with three replicates (Phillips and Hayman, 1970). Ten plants were randomly selected and harvested on day 45 after sowing for morphological measurements. Plant height and stem diameter (for the first internode) were measured using a ruler (0.01 cm accuracy) and a digital caliper (0.01 mm accuracy), respectively. The fresh weight of fresh leaves, stems and roots were weighted on 10 plants, respectively. After that, the samples were dried at 75°C for 72 h in a hot-air oven to obtain a constant dry weight to measure the relative water content.

### Measurements of Water Uptake Under Saline Conditions

Ten plants were randomly selected from each treatment to measure water uptake by plant. Each plant was considered as a biological replicate, and three measurements were carried out on each plant to obtain an average value for each biological replicate. The relative water content of leaves was measured based on: (fresh weight - dry weight)/(turgid weight – dry weight) × 100% (Zhang L. et al., 2013). The water use efficiency of watermelon leaves was determined by using a portable photosynthesis system (LI-6400, Li-Cor Inc., NE, United States).

### Measurements of Elemental Concentrations

Ten plants were randomly selected from each treatment to the elemental concentrations in plants. Each plant was considered as a biological replicate, and three measurements were carried out on each plant to obtain an average value for each biological replicate. Approximately 0.25 g of oven-dried and finely ground (<0.5 mm) sample was used to measure the contents of minerals. Total nitrogen (N) content was determined by the Kjeldahl method (Yan et al., 2012) using the Kjeldahl apparatus (Hanon K9840, Jinan, China), while total phosphorus (P), potassium (K), calcium (Ca), magnesium (Mg), Na, copper (Cu), iron (Fe), and zinc (Zn) contents were measured using the inductively coupled plasma mass spectrometer (ICP-MS, Agilent 7500ce, Agilent Technologies, CA, United States) after acidic digestion to a final solution contained 2% HNO_3_ (Chen S. et al., 2017).

### Measurement of Proline, MDA, Chlorophyll Content, Chlorophyll Fluorescence, and Chloroplast Ultrastructure

The content of proline and malondialdehyde (MDA) in watermelon leaves were measured using the acid-ninhydrin and colorimetric methods, respectively, as previously described (Cao et al., 2018). The chlorophyll (Chl) content in leaves was determined spectrophotometrically after ethanol extraction, as described by Sartory and Grobbelaar (1984). The photosynthetic rate (Pn) was determined on the second fully expanded leaves using a portable photosynthesis system (LI-6400, Li-Cor Inc., Nebraska, United States). The assimilation chamber conditions were as follows: 400 ± 10 ppm ambient CO_2_ concentration, 1000 μmol⋅m^-2^⋅s^-1^ photosynthetic photon flux density, 85% relative humidity and 25°C leaf temperature. After a 30 min dark-adaptation period, Chl fluorescence was measured with a modulated fluorimeter (PAM Photosynthesis Yield Analyzer, Walz, Effeltrich, Germany). The maximum photochemical efficiency (PE) of PSII (Fv/Fm), actual PE of PSII (Fv′/Fm′), and photochemical and non-photochemical quenching (qP and NPQ) were calculated by using equations described in Moradi and Ismail (2007). Ten plants were randomly selected from each treatment. Each plant was considered as a biological replicate, and three measurements were carried out on each plant to obtain an average value for each biological replicate. In addition, the ultrastructure of mesophyll cell chloroplasts was determined by a transmission electron microscopy (JEM-1230, JEOL, Tokyo, Japan) according to the method described in Wang et al. (2012).

### Determination of H_2_O_2_, O2^•-^ and the Activities of SOD, APX, CAT, and GR

The hydrogen peroxide (H_2_O_2_) and superoxide anion (O_2_^•-^) were assayed as described in Cao et al. (2018). Enzymatic activities were measured in leaves. The superoxide dismutase (SOD) activity was estimated by monitoring SOD-mediated inhibition of the photochemical reduction of nitro blue tetrazolium (NBT) as described in Giannopolitis and Ries (1977). The ascorbate peroxidase (APX) activity was measured by analyzing ascorbate oxidation at 290 nm wavelength (Nakano and Asada, 1987). The catalase (CAT) activity was assayed as a decrease in the absorbance at 240 nm wavelength (Bai et al., 2010). The GR activity was measured as a decrease in the absorbance at 340 nm wavelength. The monodehydroascorbate reductase (MDHAR) activity was assayed as a decrease in the absorbance at 340 nm wavelength, while the dehydroascorbate reductase (DHAR) activity was determined as an increase in the absorbance at 265 nm wavelength (Bai et al., 2010; Mo et al., 2016). Ten plants were randomly selected from each treatment. Each plant was considered as a biological replicate, and three measurements were carried out on each plant to obtain an average value for each biological replicate.

### RNA Extraction and mRNA Expression Analysis

Total RNA was extracted and purified from fresh leaves by using the RNeasy Plant Mini Kit (Takara, Dalian, China) following manufacturer’s recommendations. cDNA synthesis was performed using the SuperscriptIII First-Strand Synthesis System (Invitrogen, Shanghai, China) following the manufacturer’s protocol. Real-time PCR was performed using SYBR Premix Ex Taq (Takara, Dalian, China) in a Bio-Rad CFX96 real-time PCR system (Bio-Rad, Hercules, CA, United States). The *actin* was used as an internal control gene. The primers used are listed in Supplementary Table S1. Three plants were randomly selected from each treatment. Each plant was considered as a biological replicate, and three measurements were carried out on each plant to obtain an average value for each biological replicate.

### Statistical Analysis

For each biological replicate, the average value was obtained based on three measurements. Analysis of variance (ANOVA) was performed in the SAS statistics program (version 8.0, SAS Institute Cary, NC, United States) and R language (R Core Team, 2018). Significant tests were performed using the Duncan’s multiple range tests. Plots were generated in OriginPro (version 8.0, Origin Lab, MA, United States) and R language ggplot2 package (Wickham, 2016). Two way ANOVA test were performed in R using the aov function.

## Results

### Development of the AMF

After inoculation with AMF, the percentage of mycorrhizal colonization ratio and the number of entry points on roots were both significantly reduced compared to that under the salinity-alkalinity stresses (Table 1). Similarly, the number of arbuscular and vesicles were also both significantly decreased compared to that under the salinity-alkalinity stresses. As expected, no AMF colonization was found in the roots of the non-inoculated watermelon seedlings.

**Table 1:** Mycorrhizal colonization and fungal structures in roots of watermelon inoculated (M, MS) or non-inoculated (CK, S) seedlings with AMF *Funneliformis mosseae* and subjected (S, MS) or not (CK, M) to salinity-alkalinity stress.

Treatment	Mycorrhizal colonization ratio (%)	Number of entry points on root (Num.cm^-1^)	Number of arbuscular (Num.cm^-1^)	Number of vesicles in root (Num.cm^-1^)
CK	0.00 ± 0.00^c^	0.00 ± 0.00^c^	0.00 ± 0.00^c^	0.00 ± 0.00^c^
M	41.13 ± 1.47^a^	10.9 ± 0.67^a^	8.2 ± 0.45^a^	5.81 ± 0.26^a^
S	0.00 ± 0.00^c^	0.00 ± 0.00^c^	0.00 ± 0.00^c^	0.00 ± 0.00^c^
MS	36.22 ± 1.12^b^	7.65 ± 0.33^b^	5.8 ± 0.38^b^	2.91 ± 0.08^b^


*The values (mean ± standard error, *n* = 10) with different letters within columns are statistically different (*P* ≤ 0.05) according to Duncan’s multiple range test. AMF, arbuscular mycorrhizal fungi; CK, without AMF inoculation + without salinity-alkalinity; M, with AMF inoculation + without salinity-alkalinity; S, without AMF inoculation + with salinity-alkalinity; and MS, with AMF inoculation + with salinity-alkalinity.*

### Growth and Elemental Concentrations in Leaves of Watermelon Inoculated or Non-inoculated Seedlings With AMF and Subjected or Not to Salinity-Alkalinity Stress

Under salinity-alkalinity stresses, watermelon growth and development were significantly decreased, including plant height, stem diameter, dry weight of root and shoot (Table 2).

**Table 2:** Growth parameters of watermelon inoculated (M, MS) or non-inoculated (CK, S) seedlings with AMF *Funneliformis mosseae* and subjected (S, MS) or not (CK, M) to salinity-alkalinity stress.

Treatment	Plant height (cm)	Stem diameter (mm)	Root dry weight (g)	Shoot dry weight (g)
CK	28.13 ± 0.47^b^	3.33 ± 0.15^a,b^	1.28 ± 0.03^b^	0.18 ± 0.02^b^
M	31.73 ± 0.51^a^	3.50 ± 0.10^a^	1.41 ± 0.05^a^	0.22 ± 0.04^a^
S	16.83 ± 0.31^d^	3.03 ± 0.15^c^	0.71 ± 0.02^d^	0.10 ± 0.02^c^
MS	22.57 ± 0.78^c^	3.13 ± 0.06^b,c^	0.85 ± 0.06^c^	0.14 ± 0.03^b,c^


*The values (mean ± standard error, *n* = 10) with different letters within columns are statistically different (*P* ≤ 0.05) according to Duncan’s multiple range test. AMF, arbuscular mycorrhizal fungi; CK, without AMF inoculation + without salinity-alkalinity; M, with AMF inoculation + without salinity-alkalinity; S, without AMF inoculation + with salinity-alkalinity; and MS, with AMF inoculation + with salinity-alkalinity.*

Under salinity-alkalinity conditions, when plants were treated with AMF, the plant height, stem diameter, root dry weight, and shoot dry weight were all significantly increased compared to the non-AMF treatment. Under normal conditions with no salinity-alkalinity stresses, AMF could also significantly increase the plant height, stem diameter, root dry weight, and shoot dry weight by 12.80, 5.11, 10.16, and 22.22%, respectively, compared to the non-AMF treatment. These results demonstrated that AMF improved watermelon seedling growth under both normal and salinity-alkalinity conditions.

To study the impact of AMF colonization on watermelon seedlings, we determined the elemental uptake in the roots of plants treated by AMF-inoculated. Under salinity-alkalinity conditions, when compared to the normal control, the contents of N, P, K, Cu, Fe, and Zn content were significantly increased by 13.98, 20.08, 5.16, 9.26, and 13.27%, respectively (Table 3). In contrast, the contents of Ca, Mg, and Na were significantly decreased by 24.31, 26.91, and 26.51%, respectively. These results showed a complex impact of AMF on watermelon elemental uptake in the roots. Two-way ANOVA test showed a significant difference (*P* = 3.20 × 10^-5^) between seedlings subjected or not to salinity-alkalinity stress. Inoculation with AMF significantly (*P* < 0.05) altered the elemental uptake (Supplementary Table S2).

**Table 3:** Elemental concentrations in leaves of watermelon inoculated (M, MS) or non-inoculated (CK, S) seedlings with AMF *Funneliformis mosseae* and subjected (S, MS) or not (CK, M) to salinity-alkalinity stress.

Treatments	N (mg⋅g^-1^)	P (mg⋅g^-1^)	K (mg⋅g^-1^)	Ca (mg⋅g^-1^)	Mg (mg⋅g^-1^)	Na (mg⋅g^-1^)	Cu (μg⋅g^-1^)	Fe (μg⋅g^-1^)	Zn (μg⋅g^-1^)
CK	36.72 ± 0.05^b^	2.18 ± 0.03^c^	15.11 ± 0.03^a^	28.79 ± 0.27^a^	9.65 ± 0.07^a^	1.29 ± 0.09^c^	4.12 ± 0.06^c^	35.12 ± 0.86^a^	28.14 ± 0.06^b^
M	43.71 ± 3.10^a^	2.44 ± 0.12^b^	15.28 ± 0.09^a^	28.26 ± 0.26^a^	8.51 ± 0.01^b^	1.01 ± 0.04^d^	7.29 ± 0.16^a^	33.61 ± 0.14^a^	31.19 ± 1.71^a^
S	34.61 ± 0.30^b^	2.39 ± 0.01^b^	14.35 ± 0.09^c^	24.14 ± 0.07^b^	7.69 ± 0.06^c^	2.15 ± 0.04^a^	6.37 ± 0.15^b^	30.44 ± 0.17^b^	25.04 ± 0.05^c^
MS	39.45 ± 0.23^a^	2.87 ± 0.02^a^	15.09 ± 0.08^b^	18.27 ± 0.03^c^	5.62 ± 0.05^d^	1.58 ± 0.13^b^	6.96 ± 0.04^a^	34.48 ± 0.16^a^	29.21 ± 0.09^b^


*The values (mean ± standard error, *n* = 10) with different letters within columns are statistically different (*P* ≤ 0.05) according to Duncan’s multiple range test. AMF, arbuscular mycorrhizal fungi; CK, without AMF inoculation + without salinity-alkalinity; M, with AMF inoculation + without salinity-alkalinity; S, without AMF inoculation + with salinity-alkalinity; and MS, with AMF inoculation + with salinity-alkalinity.*

### Leaf Water Uptake of Watermelon Inoculated or Non-inoculated Seedlings With AMF and Subjected or Not to Salinity-Alkalinity Stress

**FIGURE 1:**
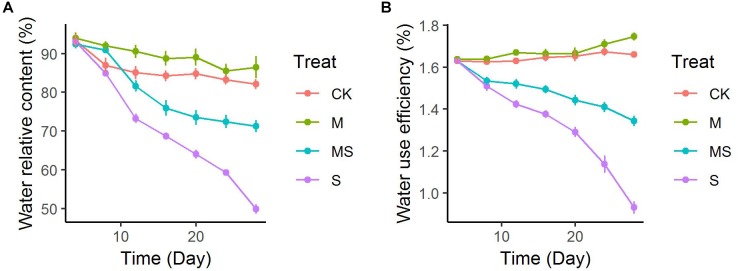
Leaf water uptake of watermelon inoculated or non-inoculated seedlings with AMF *Funneliformis mosseae* and subjected or not to salinity-alkalinity stress from 4 to 28 days. **(A)** Water relative content; **(B)** water use efficiency. AMF, arbuscular mycorrhizal fungi; CK, without AM inoculation + without salinity-alkalinity; M, with AM inoculation + without salinity-alkalinity; S, without AM inoculation + with salinity-alkalinity; MS, with AM inoculation + with salinity-alkalinity. Values are means ± standard errors (*n* = 10).

The leaf relative water content was decreased gradually in seedlings both inoculated or non-inoculated with AMF and subjected or not to salinity-alkalinity stresses (Figure 1A). After 28 days of treatment, leaf relative water content under salinity-alkalinity stress was decreased from approximately 93 to 49.86%, which was significantly alleviated to 71.26%. Two-way ANOVA test showed there was a significant difference (*P* = 9.78 × 10^-11^) between seedlings subjected or not to salinity-alkalinity stress. Inoculation with AMF significantly (*P* = 3.20 × 10^-5^) altered the leaf relative water content (Supplementary Table S3). The alleviation effects of AMF under salinity-alkalinity stresses reached plateau after 20 days’ treatment. Notably, even when plants did not suffer from salinity-alkalinity stresses (CK), AMF increased the water relative content from 82.12 to 86.48%. Compared to normal conditions, salinity-alkalinity stress significantly reduced the water use efficiency (Figure 1B). After 28 days of treatment, AMF inoculation under no salinity-alkalinity stress also increased the water use efficiency from 1.66 to 1.75% (Figure 1B). When plants were under salinity-alkalinity stress, AMF inoculation significantly alleviated the water use efficiency from 0.93 to 1.34% (Figure 1B). Two-way ANOVA test revealed similar result compared to that of leaf relative water content (Supplementary Table S4).

### ROS Concentrations and ROS Scavenging Enzymes in Leaves of Watermelon Seedlings Inoculated or Non-inoculated With AMF and Subjected or Not to Salinity-Alkalinity Stress

**FIGURE 2:**
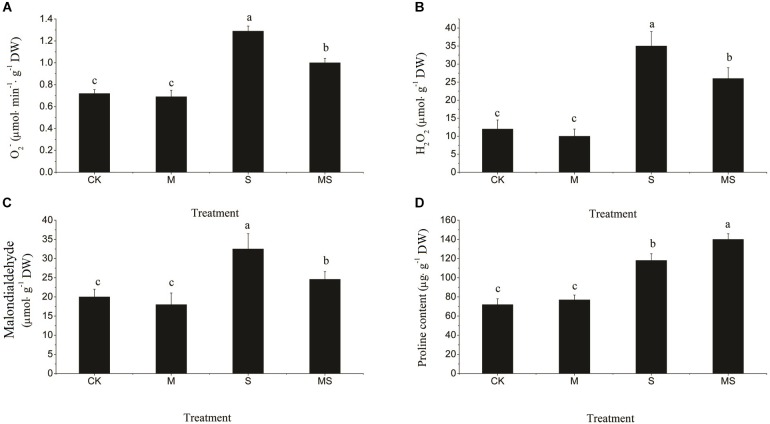
Reactive oxygen species (O_2_^•-^ and H_2_O_2_), MDA, and proline content in watermelon seedlings in leaves of watermelon inoculated or non-inoculated seedlings with AMF *Funneliformis mosseae* and subjected or not to salinity-alkalinity stress. **(A)** O_2_^•-^ content; **(B)** H_2_O_2_ content; **(C)** MDA content; (**D**) Proline content. ROS, relative oxygen species; O_2_^•-^, superoxide anion; H_2_O_2_, hydrogen peroxide; MDA, malondialdehyde; AMF, arbuscular mycorrhizal fungi; CK, without AM inoculation + without salinity-alkalinity; M, with AM inoculation + without salinity-alkalinity; S, without AM inoculation + with salinity-alkalinity; and MS, with AM inoculation + with salinity-alkalinity. Values are means ± standard errors (*n* = 10). Bars with different letters are significantly different at the 0.05 level (Duncan’s multiple range test).

Salinity-alkalinity stresses significantly increased the contents of O_2_^•-^ and H_2_O_2_ (Figure 2A,B). After AMF treatment, the accumulation of O_2_^•-^ and H_2_O_2_ were both significantly reduced compared with the non-AMF inoculated treatment. MDA, which can damage the cell membrane, was significantly increased by salinity-alkalinity stress compared to the control, which was significantly decreased after AMF treatment (Figure 2C). An increased accumulation of proline in the leaves subjected to stress indicated an effective plant stress response at the metabolic level. Salinity-alkalinity stresses significantly increased the content of proline. However, proline was accumulated in the greatest quantities under the AMF inoculated plants in the salinity-alkalinity treatment (Figure 2D).

**FIGURE 3:**
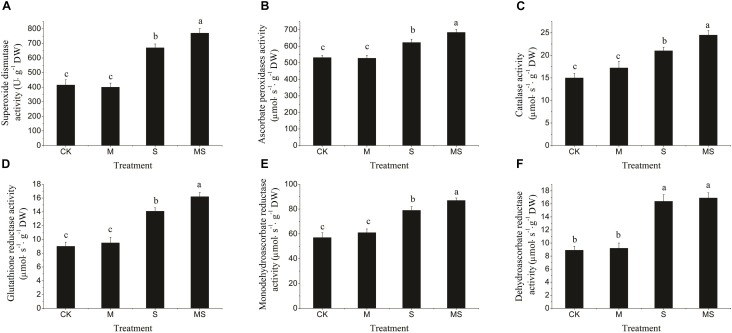
Reactive oxygen species scavenging enzymes (SOD, APX, CAT, GR, MDHAR, and DHAR) in watermelon inoculated or non-inoculated seedlings with AMF *Funneliformis mosseae* and subjected or not to salinity-alkalinity stress. **(A)** SOD activity; **(B)** APX activity; **(C)** CAT activity; **(D)** GR activity; **(E)** MDHAR activity; **(F)** DHAR activity. ROS, relative oxygen species; SOD, superoxide dismutase; APX, ascorbate peroxidases; CAT, catalase; GR, glutathione reductase; MDHAR, monodehydroascorbate reductase; DHAR, dehydroascorbate reductase; AMF, arbuscular mycorrhizal fungi; CK, without AM inoculation + without salinity-alkalinity; M, with AM inoculation + without salinity-alkalinity; S, without AM inoculation + with salinity-alkalinity; and MS, with AM inoculation + with salinity-alkalinity. Values are means ± standard errors (*n* = 10). Bars with different letters are significantly different at the 0.05 level (Duncan’s multiple range test).

In this experiment, the activities of SOD, APX, CAT, GR, MDHAR, and DHAR enzymes were significantly increased in response to the salinity-alkalinity treatment, which were further significantly increased after the AMF treatment (Figure 3). When there was no salinity-alkalinity stress, AMF treatment had no significant influence on the activities of the enzymes aforementioned (Figure 3). These results revealed that watermelon seedlings inoculated with AMF grown under salinity-alkalinity conditions improved the tolerance to salinity-alkalinity stresses. Two-way ANOVA test showed that salinity-alkalinity treatment had a significant (*P* = 7.35 × 10^-9^) impact on the ROS related enzyme activities. However, Inoculation with AMF had no significant impact when taking all enzymes together (Supplementary Table S5).

### Photosynthetic Pigments in Watermelon Inoculated or Non-inoculated Seedlings With AMF and Subjected or Not to Salinity-Alkalinity Stress

**FIGURE 4:**
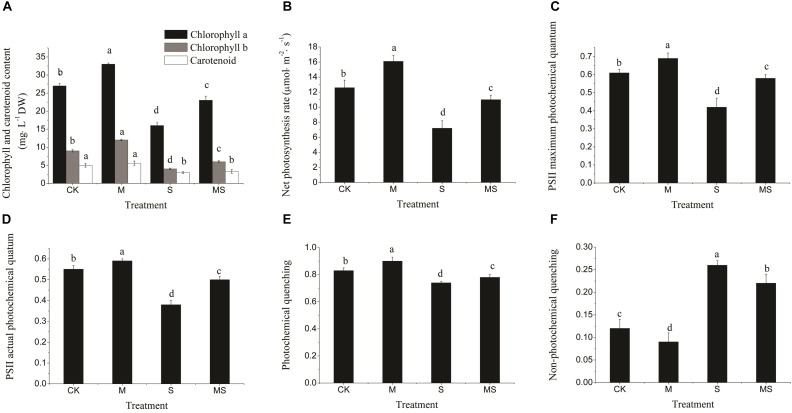
Chlorophyll content, photosynthesis rate, and PSII electron transport in watermelon inoculated or non-inoculated seedlings with AMF *Funneliformis mosseae* and subjected or not to salinity-alkalinity stress. **(A)** Chlorophyll a, chlorophyll b, and carotenoid content. **(B)** Net photosynthesis rate. **(C)** PSII maximum photochemical quantum. **(D)** PSII actual photochemical quantum. **(E)** Photochemical quenching. **(F)** Non-photochemical quenching. PSII, photosynthesis system II; AMF, arbuscular mycorrhizal fungi; CK, without AM inoculation + without salinity-alkalinity; M, with AM inoculation + without salinity-alkalinity; S, without AM inoculation + with salinity-alkalinity; and MS, with AM inoculation + with salinity-alkalinity. Values are means ± standard errors (*n* = 10). Bars with different letters are significantly different at the 0.05 level (Duncan’s multiple range test).

Under normal conditions, all photosynthesis-related parameters were maintained at relatively high levels, which were all significantly improved after further add-in of AMF, including chlorophyll and carotenoid contents, net photosynthesis rate, PSII maximum, actual photochemical quantum and photochemical quenching (Figure 4A–E). In contrast, the non-photochemical quenching was significantly reduced (Figure 4F). Under salinity-alkalinity stresses, all photosynthesis-related parameters were significantly reduced compared to normal conditions and were significantly alleviated after further add-in of AMF (Figure 4). Taken together, these results demonstrated that AMF could not only alleviate the impacts of salinity-alkalinity stresses, but also promote the photosynthesis-related processes under normal conditions.

**FIGURE 5:**
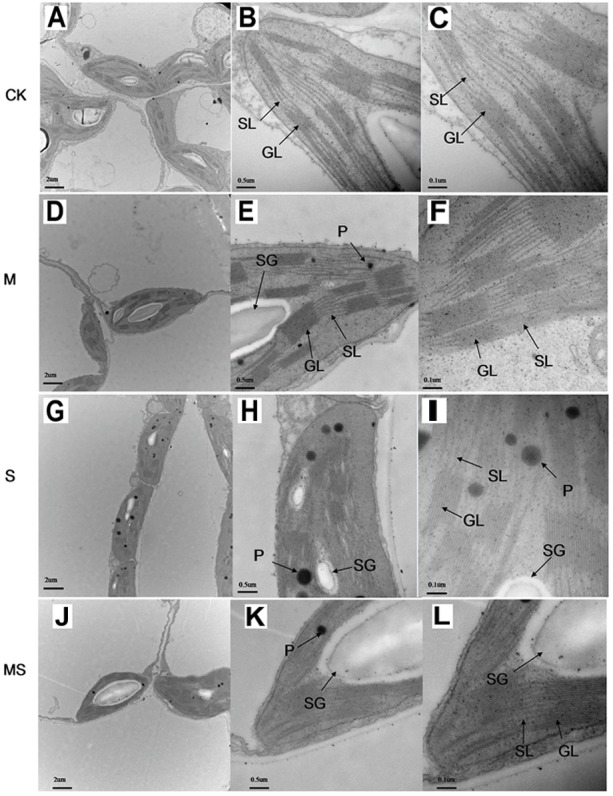
Chloroplast ultrastructure in watermelon inoculated or non-inoculated seedlings with AMF *Funneliformis mosseae* and subjected or not to salinity-alkalinity stress. **(A)** Mesophyll cells in CK; **(B)** chloroplast in CK; **(C)** thylakoid in CK. **(D)**; mesophyll cells in M; **(E)** chloroplast in M; **(F)** chloroplast in M; **(G)** mesophyll cells in S; **(H)** chloroplast in S; **(I)** thylakoid in S; **(J)** mesophyll cells in MS; **(K)** chloroplast in MS; **(L)** chloroplast in MS. AMF, arbuscular mycorrhizal fungi; CK, without AM inoculation + without salinity-alkalinity; M, with AM inoculation + without salinity-alkalinity; S, without AM inoculation + with salinity-alkalinity; and MS, with AM inoculation + with salinity-alkalinity. Data were obtained from the second expanded leaves (numbered basipetally) after salinity-alkalinity treatment for 8 days. SL, stroma lamellae; GL, grana lamellae; SG, starch grains; P, plastoglobuli. Scale bars for chloroplasts and thylakoids are 2, 0.5, and 0.1 μm, respectively.

A decrease in the net photosynthesis rate reflects the degradation of pigments in the chloroplast. We then characterized the chloroplast ultrastructure of the watermelon leaves. Typical spindle chloroplasts were observed in watermelon seedlings in the CK and M treatments, with intact double membranes and a regular arrangement of grana and stromal thylakoids (Figure 5A–F). Under salinity-alkalinity stresses, the chloroplast structure of watermelon seedlings were damaged; the stroma thylakoid were stacked, the grana were blurred; the chloroplasts were swollen; the lamellar structure was destroyed; the number of plastoglobuli were increased; and the plastoglobule volume was abnormally large (Figure 5G–I). The extent of damage on the chloroplast structure of watermelon seedlings grown under salinity-alkalinity stress was significantly alleviated by AMF treatment. Specifically, some stroma and grana thylakoid structures remained completely intact, with more normal chloroplast ultrastructure, fewer plastoglobuli, and lower plastoglobular volume (Figure 5J–L).

### Relative Expression of Stress Responsive Genes

**FIGURE 6:**
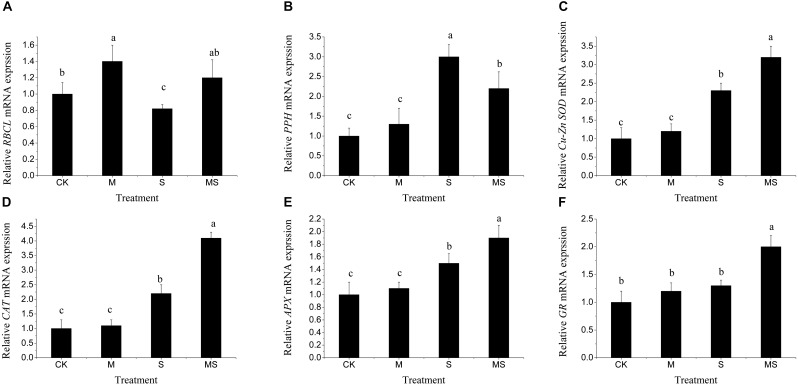
Relative expression patters of the *RBCL* **(A)**, *PPH* **(B)**, *Cu-Zn SOD* **(C)**, *CAT* **(D)**, *APX* **(E)**, and *GR* **(F)** genes in watermelon inoculated or non-inoculated seedlings with AMF *Funneliformis mosseae* and subjected or not to salinity-alkalinity stress. Values are means ± standard errors (*n* = 3). Bars with different letters are significantly different at the 0.05 level (Duncan’s multiple range test). *RBCL*, rubisco large subunit; *PPH*, pheophytin pheophorbide hydrolase; *Cu-Zn SOD*, Cu-Zn subunit-superoxide dismutase; *CAT*, Catalase; *APX*, cytoplasmic ascorbate peroxidase; *GR*, cytoplasmic glutathione reductase; AMF, arbuscular mycorrhizal fungi.

To explore the molecular regulations of watermelon seedlings in the AMF treatment under salinity-alkalinity stresses, the expressions of genes involved in photosynthesis (*RBCL*), chlorophyll degradation (*PPH*), and antioxidant response (*Cu-Zn SOD, CAT, APX, GR*) were analyzed by using qRT-PCR. Under normal conditions, AMF significantly increased the relative expression level of *RBCL*. Under salinity-alkalinity stresses, the relative expression level of *RBCL* was significantly reduced compared to that under normal conditions and were significantly alleviated after AMF treatment (Figure 6A). For chlorophyll degradation related gene PPH, under normal conditions, AMF also increased the relative expression level of *PPH*, but not significantly. Under salinity-alkalinity stresses, the relative expression level of *PPH* was significantly increased and was further significantly reduced after AMF (Figure 6B). For the relative expression level of antioxidant response related genes *Cu-Zn SOD, CAT, APX, GR*, there was no significant difference between normal conditions and single AMF treatment (Figure 6C–F). Under salinity-alkalinity stresses, their relative expression levels were significantly increased and were further significantly increased with AMF treatment (Figure 6C–F). These results demonstrated that AMF could significantly improve the antioxidant responses under salinity-alkalinity stresses. Two-way ANOVA test showed that salinity-alkalinity stress had a significant impact on these genes (Supplementary Table S6). Though inoculating or non-inoculating AMF had no significant impact on all these genes (*P* > 0.05), for each gene, it significantly (*P* < 0.001) altered the expression levels (data not shown).

## Discussion

Arbuscular mycorrhizal fungi establishes mutualistic interactions with more than 80% of all plant species, providing a direct physical link between soils and plant roots to reach a regional nutrient-rich zone (Coleman-Derr and Tringe, 2014; Lenoir et al., 2016). AMFs have been implicated in boosting plant growth, photosynthesis, and tolerance to biotic and abiotic stresses (Cavagnaro et al., 2015; Liu et al., 2016). Nevertheless, the knowledge about the regulatory mechanisms underlying AMF-mediated tolerance under salinity-alkalinity stresses still remain fragmentary and limited. In this study, we examined the effects of AMF treatment on plant growth, photosynthesis, redox state, and chloroplast ultrastructure in watermelon seedlings under salinity-alkalinity stresses. Our results indicated that inoculation with AMF could enhance the tolerance of salinity-alkalinity stresses of watermelon seedlings by improving photosynthesis, water use efficiency, preventing damage to the chloroplast ultrastructure, and enhancing cellular redox reactions.

Promotion of plant growth by AMF has been documented in some plant species (Huang et al., 2011; Mo et al., 2016). In this study, we found that AMF had a positive effect on plant height, stem diameter, root dry weight, and shoot dry weight of watermelon seedlings, particularly under salinity-alkalinity stress conditions (Table 2). Even when plants were under no salinity-alkalinity stresses, AMF could also promote plant growth and development of some aspects, which can be caused by other microorganisms, such as bacteria that exerted some effects on plant physiology (Azcón-Aguilar and Barea, 1997). Water relative content and water use efficiency were both significantly alleviated after AMF treatment under salinity-alkalinity stress conditions. Through the large hyphal network of AMF in the soil, extraradical mycelium absorbs more nutrients, transports nutrients into the fungal intraradical mycelium, and releases nutrients at arbuscules, allowing roots of watermelon seedlings to absorb more water and nutrients (Karandashov and Bucher, 2005; Bonfante and Genre, 2010; Zhang N. et al., 2013; Lenoir et al., 2016; Hashem et al., 2018). This might explain why under salinity-alkalinity stress conditions, the watermelon seedlings inoculated with AMF had a better growth status than the non-inoculated seedlings.

Plants can improve their tolerance to salinity-alkalinity stress through multiple biochemical pathways, such as production of osmotically dynamic metabolites, free radicals, and specific proteins that manage ion and water flux (Ahmad et al., 2008; Li et al., 2015). Proline can stabilize the subcellular structures and scavenge free radicals, and MDA is the decomposition product of polyunsaturated fatty acids of membranes and is used to assess the severity of oxidative damage (Huang et al., 2015; Li et al., 2015; Cao et al., 2018). Our results indicated that plants inoculated with AMF and grown under salinity-alkalinity conditions had a decreased concentration of MDA and an increased concentration of proline (Figure 1). The enhanced production of ROS is a typical stress-derived physiological response due to the inefficient dissipation of excessive excitation energy caused by damage of the photosynthetic apparatus, which is harmful to cells and results in oxidative damage (Fan and Liu, 2011; Noctor et al., 2014; Mo et al., 2016). In this study, the accumulation of H_2_O_2_ and O_2_^•-^ under salinity-alkalinity stress was decreased when watermelon seedlings were inoculated with AMF. ROS scavenging is important to alleviate such oxidative stresses and to maintain normal plant metabolism (Miller et al., 2010; Noctor et al., 2014; Mo et al., 2016; Pedranzani et al., 2016). Enzymatic antioxidants, such as SOD, CAT, APX, GR, MDHAR, and DHAR, can effectively alleviate the excess ROS, maintaining the balance of the formation and removal of ROS (Miller et al., 2010; Mo et al., 2016). An increase in the activity of antioxidant enzymes and gene expressions of SOD, CAT, APX, GR, MDHAR, and DHAR were found in the plants inoculated with AMF under salinity-alkalinity conditions (Figure 2), suggesting that AMF induced an efficient ROS scavenging mechanism to protect the watermelon seedlings from extensive oxidative damage. Inoculation with AMF significantly increased the enzyme activities and gene expressions involved in ROS homeostasis under abiotic stresses, providing the host plant better protection abilities against oxidative stress. These responses were correlated with plant tolerance to abiotic stress (Fan and Liu, 2011; Mo et al., 2016).

Salinity-alkalinity stresses inhibited photosynthesis and biomass accumulation in watermelon seedlings (Li et al., 2017). Drop irrigation systems with saline water could improve the yield and quality of watermelons (Lei et al., 2003). However, watermelon seedlings inoculated with AMF could alleviate salinity-alkalinity induced inhibition of photosynthesis and biomass production (Figure 3). Photon flux is absorbed by the antenna pigments that excite chlorophyll, and chlorophyll content (Shamshiri and Fattahi, 2016). A decrease in the chlorophyll content might lead to a decreased net photosynthesis rate (Figure 3). The parameters of Fv/Fm, Fv’/Fm’, qP, and NPQ have been used extensively to examine the photosynthetic efficiency of different plants subjected to different abiotic stresses (Li et al., 2015; Shamshiri and Fattahi, 2016). In this study, the levels of Fv/Fm, Fv’/Fm’, and qP, under salinity-alkalinity stresses were significantly decreased, indicating that light energy absorption and electron transport in PS II were restricted by salinity-alkalinity stresses. Additionally, higher NPQ in salinity-alkalinity stressed watermelon seedlings also indicated that excitation energy was excessive for the capacity of electron transport (Li et al., 2017). However, these decreases of Fv/Fm, Fv’/Fm’, qP, and the increase of NPQ were significantly alleviated after further treatment with AMF. Maintenance of the structural integrity of chloroplasts is very important for plants to better maintain their photosynthetic capacity (Hu et al., 2014; Mo et al., 2016). In the present study, salinity-alkalinity stress induced destruction of the chloroplast envelope and increased the number of plastoglobuli and aberrations in the thylakoid membrane. However, fewer negative impacts were observed in watermelon plants treated with AMF. Watermelon seedlings exposed to salinity-alkalinity stresses showed stunted growth compared to those seedlings inoculated with AMF. Overall, seedlings inoculated with AMF had improved tolerance to salinity-alkalinity stresses via different physiological and regulatory aspects.

## Conclusion

Arbuscular mycorrhizal fungi treatment could significantly improve watermelon nutrient uptake through a large hyphal network and alleviate salinity-alkalinity induced oxidative damage on watermelon seedlings, most likely through the regulation of antioxidant enzymes and non-enzymatic systems. Under salinity-alkalinity stresses, seedlings inoculated with AMF significantly increased the net photosynthesis rate through the maintenance of the normal chloroplast ultrastructure. After treatment with AMF, there was a significant improvement in PSII electron transport, which promoted the growth of watermelon seedlings under salinity-alkalinity stress. Therefore, these results demonstrated the benefits of AMF under salinity-alkalinity stresses, which had wide application potential in watermelon production, especially in regions with salinity-alkalinity conditions.

## Author Contributions

ZZ and KC conceived the study. LY designed the experiments. LY and XZ conducted the experiments and the data analysis. LY, KC, and EB wrote and revised the manuscript. All authors read and approved the final version of the manuscript.

## Conflict of Interest Statement

The authors declare that the research was conducted in the absence of any commercial or financial relationships that could be construed as a potential conflict of interest.
