# Supplementary material for: Effects of Arbuscular Mycorrhizal Fungi on Watermelon Growth, Elemental Uptake, Antioxidant, and Photosystem II Activities and Stress-Response Gene Expressions Under Salinity-Alkalinity Stresses
Source: Front Plant Sci. 2019 Jul 3;10:863. doi: 10.3389/fpls.2019.00863 (PMC6616249; doi:10.3389/fpls.2019.00863)
Supplement: Supplementary file 1 [file Table_1.DOC]

**Table S1.** Sequences of primers used in this study for quantitative RT-PCR.

| Gene | Gene ID | Primer sequence (5’-3’) | Gene description |
| --- | --- | --- | --- |
| RBCL | Cla003384 | F:CACCACAAACAGAGACTAAAGCA | Rubisco large subunit |
|  |  | R: TCGGAATGCTGCCAAGATAT |  |
| PPH | Cla015204 | F: GATATGGGACGTGATTACAGAG | Pheophytin pheophorbide hydrolase |
|  |  | R: GATGCTGCCATAAGTCAATAGAG |  |
| Cu-Zn SOD | Cla008698 | F: AGCCATTGTAGATACCCAGATTC | Cu-Zn subunit-superoxide dismutase |
|  |  | R: CTGAGTTCGTGACCTCCTTT |  |
| CAT | Cla023447 | F: ACTTGTGCCGATTTCCTTCG | Catalase |
|  |  | R: ATTGCCCTCCCTGGTGTAA |  |
| APX | Cla022327 | F: GGGAAGTTGAACGGATTAGAG | Cytoplasmic ascorbate peroxidase |
|  |  | R: CAGCATCGTGAAATACCAGAC |  |
| GR | Cla021430 | F: GGAGTCGCTGTCGCTCTTAA | Cytoplasmic glutathione reductase |
|  |  | R: CTACCTGCTTCAATTCGCCT |  |
