# Supplementary material for: Effects of Arbuscular Mycorrhizal Fungi on Watermelon Growth, Elemental Uptake, Antioxidant, and Photosystem II Activities and Stress-Response Gene Expressions Under Salinity-Alkalinity Stresses
Source: Front Plant Sci. 2019 Jul 3;10:863. doi: 10.3389/fpls.2019.00863 (PMC6616249; doi:10.3389/fpls.2019.00863)
Supplement: Supplementary file 2 [file Table_2.DOCX]

**Table S2.** Two-way ANOVA test of mineral concentrations in leaves of watermelon inoculated or non-inoculated seedlings with AMF and subjected or not to salinity-alkalinity stress

|  | Df | Sum Sq | Mean Sq | F value | Pr(>F) |  |
| --- | --- | --- | --- | --- | --- | --- |
| Treat2 | 1 | 23 | 23.2 | 4.784 | 0.0312 | * |
| Treat1 | 1 | 92 | 92.4 | 19.06 | 3.20E-05 | *** |
| Element | 8 | 16106 | 2013.2 | 415.069 | < 2e-16 | *** |
| Treat2:Treat1 | 1 | 1 | 1 | 0.21 | 0.6474 |  |
| Residuals | 96 | 466 | 4.9 |  |  |  |

Treat 1: Subjected or not to salinity-alkalinity stress.

Treat 2: Inoculated or not with AMF.

*** 0.001; ** 0.01; 0.01 *
