# Supplementary material for: Effects of Arbuscular Mycorrhizal Fungi on Watermelon Growth, Elemental Uptake, Antioxidant, and Photosystem II Activities and Stress-Response Gene Expressions Under Salinity-Alkalinity Stresses
Source: Front Plant Sci. 2019 Jul 3;10:863. doi: 10.3389/fpls.2019.00863 (PMC6616249; doi:10.3389/fpls.2019.00863)
Supplement: Supplementary file 3 [file Table_3.DOCX]

**Table S3.** Two-way ANOVA test of leaf relative water content in leaves of watermelon inoculated or non-inoculated seedlings with AMF and subjected or not to salinity-alkalinity stress

|  | Df | Sum Sq | Mean Sq | F value | Pr(>F) |  |
| --- | --- | --- | --- | --- | --- | --- |
| Treat2 | 1 | 896 | 896 | 15.497 | 0.000185 | *** |
| Treat1 | 1 | 3283 | 3283 | 56.773 | 9.78E-11 | *** |
| Day | 6 | 1588 | 265 | 4.577 | 0.00053 | *** |
| Treat2:Treat1 | 1 | 158 | 158 | 2.735 | 0.102425 |  |
| Residuals | 74 | 4279 | 58 |  |  |  |

Treat 1: Subjected or not to salinity-alkalinity stress.

Treat 2: Inoculated or not with AMF.

*** 0.001; ** 0.01; 0.01 *
