# Supplementary material for: Effects of Arbuscular Mycorrhizal Fungi on Watermelon Growth, Elemental Uptake, Antioxidant, and Photosystem II Activities and Stress-Response Gene Expressions Under Salinity-Alkalinity Stresses
Source: Front Plant Sci. 2019 Jul 3;10:863. doi: 10.3389/fpls.2019.00863 (PMC6616249; doi:10.3389/fpls.2019.00863)
Supplement: Supplementary file 4 [file Table_4.DOCX]

**Table S4.** Two-way ANOVA test of water use efficiency in leaves of watermelon inoculated or non-inoculated seedlings with AMF and subjected or not to salinity-alkalinity stress

|  | Df | Sum Sq | Mean Sq | F value | Pr(>F) |  |
| --- | --- | --- | --- | --- | --- | --- |
| Treat2 | 1 | 0.1783 | 0.1783 | 12.009 | 0.000884 | *** |
| Treat1 | 1 | 1.3757 | 1.3757 | 92.666 | 1.10E-14 | *** |
| Day | 6 | 0.1056 | 0.0176 | 1.186 | 0.323261 |  |
| Treat2:Treat1 | 1 | 0.0799 | 0.0799 | 5.379 | 0.023143 | * |
| Residuals | 74 | 1.0986 | 0.0148 |  |  |  |

Treat 1: Subjected or not to salinity-alkalinity stress.

Treat 2: Inoculated or not with AMF.

*** 0.001; ** 0.01; 0.01 *
