# Supplementary material for: Effects of Arbuscular Mycorrhizal Fungi on Watermelon Growth, Elemental Uptake, Antioxidant, and Photosystem II Activities and Stress-Response Gene Expressions Under Salinity-Alkalinity Stresses
Source: Front Plant Sci. 2019 Jul 3;10:863. doi: 10.3389/fpls.2019.00863 (PMC6616249; doi:10.3389/fpls.2019.00863)
Supplement: Supplementary file 5 [file Table_5.DOCX]

**Table S5.** Two-way ANOVA test of ROS scavenging enzymes in leaves of watermelon inoculated or non-inoculated seedlings with AMF and subjected or not to salinity-alkalinity stress

|  | Df | Sum Sq | Mean Sq | F value | Pr(>F) |  |
| --- | --- | --- | --- | --- | --- | --- |
| Treat2 | 1 | 1790 | 1790 | 0.836 | 0.363 |  |
| Treat1 | 1 | 84508 | 84508 | 39.462 | 7.35E-09 | *** |
| Types | 9 | 5620756 | 624528 | 291.632 | < 2e-16 | *** |
| Treat2:Treat1 | 1 | 1047 | 1047 | 0.489 | 0.486 |  |
| Residuals | 107 | 229140 | 2141 |  |  |  |

Treat 1: Subjected or not to salinity-alkalinity stress.

Treat 2: Inoculated or not with AMF.

Types: Types of ROS measured (related to Figure 2 and 3).

*** 0.001; ** 0.01; 0.01 *
