# Supplementary material for: Effects of Arbuscular Mycorrhizal Fungi on Watermelon Growth, Elemental Uptake, Antioxidant, and Photosystem II Activities and Stress-Response Gene Expressions Under Salinity-Alkalinity Stresses
Source: Front Plant Sci. 2019 Jul 3;10:863. doi: 10.3389/fpls.2019.00863 (PMC6616249; doi:10.3389/fpls.2019.00863)
Supplement: Supplementary file 6 [file Table_6.DOCX]

**Table S6.** Two-way ANOVA test of relative expression of stress responsive genes in leaves of watermelon inoculated or non-inoculated seedlings with AMF and subjected or not to salinity-alkalinity stress

|  | Df | Sum Sq | Mean Sq | F value | Pr(>F) |  |
| --- | --- | --- | --- | --- | --- | --- |
| Treat2 | 1 | 0.41 | 0.41 | 0.411 | 0.524 |  |
| Treat1 | 1 | 31.01 | 31.01 | 30.876 | 5.93E-07 | *** |
| Genes | 5 | 161.75 | 32.35 | 32.213 | 3.84E-16 | *** |
| Treat2:Treat1 | 1 | 0.11 | 0.11 | 0.108 | 0.744 |  |
| Residuals | 63 | 63.27 | 1 |  |  |  |

Treat 1: Subjected or not to salinity-alkalinity stress.

Treat 2: Inoculated or not with AMF.

Types: Types of ROS measured (related to Figure 2 and 3).

*** 0.001; ** 0.01; 0.01 *
